# Comparison of Rally Length between Women and Men in High-Level Spanish Volleyball

**DOI:** 10.5114/jhk/167053

**Published:** 2023-07-06

**Authors:** Raúl Hileno, Marc Gonzàlez-Franqué, Albert Iricibar, Lorenzo Laporta, Antonio García-de-Alcaraz

**Affiliations:** 1 National Institute of Physical Education of Catalonia, University of Lleida, Lleida, Spain.; 2 Núcleo de Estudos em Performance Analysis em Esportes (NEPAE/UFSM), Centro de Educação Física e Desportos da Universidade Federal de Santa Maria, Santa Maria, Brazil.; 3 Faculty of Educational Sciences, University of Almería, Almería, Spain.

**Keywords:** match analysis, team sports, gender, non-parametric statistics, regression analysis, survival analysis

## Abstract

The aim of this study was to evaluate whether the rally length in high-level Spanish volleyball was longer in women than in men. A total of 1,786 rallies were observed: 792 for women and 994 for men. The recorded variables were match (quarter-final 1, quarter-final 2, semi-final 1, semi-final 2, final), gender (men, women), rally length (seconds), pseudo-rally (no, yes), and terminal event (ball out of sight, ball in/out, fault). Different non-parametric statistical techniques were used to compare the rally length between groups or subsets of data, i.e., the Kruskal-Wallis H test, the Mann-Whitney U test, quantile regression, and survival analysis. The mean and median rally length was significantly and slightly longer in women than in men. The rally length difference between genders was barely 1 s in quantile 0.5 or median, while in quantile 0.95, it was just over 4 s. In women, the probability of ending the rally at 3.9, 5.1, 10.2, and 43.9 s (at 4.4, 6.3, 11.6, and 43.9 s without pseudo-rallies) was 25%, 50%, 75%, and 100%, respectively. In men, the probability of ending the rally at 3.2, 4.3, 7.9, and 29.1 s (at 3.9, 4.8, 8.8, and 29.1 s without pseudo-rallies) was 25%, 50%, 75%, and 100%, respectively. These temporal thresholds can help volleyball coaches to train their players in a coherent manner.

## Introduction

Since its foundation, the Fédération Internationale de Volleyball (FIVB) has made multiple changes to the rules of the game to balance the attack-defence relationship and to increase the rally length and the entertainment of spectators. For example, changes such as the introduction of the libero player in 1998 were created to evolve the game in terms of rally length and multi-phase play (FIVB, 2016). However, João et al. (2006) found that liberos, compared to outside hitters, increased the reception quality and the attack effectiveness in Complex I and, consequently, decreased game continuity. Therefore, these changes in rules of the game have not always had the desired effect for the FIVB.

The official volleyball rules (FIVB, 2021) define a rally as a sequence of game actions that lasts from the hit of the server until the ball is out of play (i.e., until the referee whistles the end of the rally). The rules of the game also indicate different ways to end a rally, which can be classified into two groups: (a) rallies ending with a ball in or out (e.g., ball that touches the floor, the ceiling, the antennae, the posts, an object outside the court, a person out of play, etc.); or (b) rallies ending with a fault committed by a team before the ball is in or out (e.g., double contact fault, four hits fault, catch fault, contact with the net fault, etc.). On the other hand, several researchers specialised in volleyball define the rally length as the time in seconds that elapses from the hit of the server to the last hit that occurs just before the referee whistles the end of the rally (Aytar et al., 2019; Sánchez-Moreno et al., 2015, 2016). In addition to these temporal limits, these researchers propose more specific ones for situations in which the serve is failed or in which the ball is out of sight because it goes out of the frame captured by the video camera lens.

In relation to the rally length in the senior category, recent studies analysed its mean and standard deviation (*SD*), despite the fact that this continuous variable did not follow a normal distribution (Aytar et al., 2019; García-de-Alcaraz et al., 2017; Mroczek and Pawlik 2023; Sánchez-Moreno et al., 2015, 2016). There are also several studies from the seventies and eighties (e.g., Dyba, 1982; Lecompte and Rivet, 1979; Viitasalo et al., 1987) that have not been considered because there have been many regulatory changes since then, such as the scoring system or the incorporation of the libero player. In senior women, Aytar et al. (2019) found that the mean rally length was 6.88 s (*SD* = 5.94) in the Turkish top-level league. In senior men, Sánchez-Moreno et al. (2015, 2016) detected that the mean rally length was 4.99 s (*SD* = 4.35) in several international competitions (2010 World Championship, 2010 and 2011 World League, 2011 European Championship, and 2011 European League); and García-de-Alcaraz et al. (2017) observed that it was 5.84 s (*SD* = 4.23) and 6.79 s (*SD* = 4.71) in a national (2008–09 and 2009–10 Spanish Superliga) and an international competition (2008 Olympic Games), respectively. From those results, a hypothesis can be deduced that in high-level senior volleyball, the mean rally length and its dispersion is slightly greater in women than in men. However, those studies had the limitation that they did not analyse both genders together, using common criteria to measure the rally length or appropriate statistical techniques to compare them. To our knowledge, only one sitting volleyball study has analysed both genders together (Tsakiri et al., 2021), but contrary to our hypothesis, this study found that men’s rallies (*M* = 5.98 s, *SD* = 4.4) lasted longer than women’s rallies (*M* = 4.98 s, *SD* = 3.5) in a senior international competition (2019 European Sitting Volleyball Championship)

Regarding the rally length in the youth and junior category, García-de-Alcaraz et al. (2017) found in a Spanish men’s national championship that the average duration was 8.91 s (*SD* = 7.36), 8.34 s (*SD* = 6.64), and 7.58 s (*SD* = 5.73) in Under 14, Under 16, and Under 19, respectively. From those results, it can be concluded that as the age category in men increases, the mean rally length and its dispersion decrease. Possibly, this hypothesis can also be formulated in women from the youth and junior categories. However, no previous studies have been found to confirm it.

For its part, since 2006, the FIVB Rules of the Game & Refereeing Commission has developed a scientific research project, called *Picture of the Game*, to inform the international volleyball community about numerical aspects of the game, such as the average rally duration or the average number of ball contacts during one rally. In the latest report published by this commission (FIVB, 2019), the 2019 FIVB Volleyball Nations League final round was analysed, and a mean rally length of 7.13 s in women and 5.65 s in men was found (mean difference = 1.48 s). A disadvantage is that this report did not specify the methodology used to record and analyse the data. However, an advantage is that it did introduce the concept of *pseudo-rallies* in its analyses (i.e., rallies that ended very soon due to a serve error or an ace). Thus, when pseudo-rallies were removed from the analysis, the mean rally length was 8.31 s for women and 7.10 s for men (mean difference = 1.21 s). This concept of pseudo-rallies was also introduced by Aytar et al. (2019), but, in contrast, those researchers did not take it into account in their analyses.

Therefore, considering the main methodological limitations detected in previous studies on rally length (i.e., analysing women and men separately without distinguishing the pseudo-rallies or the event that ends the rally, or analysing rally length with inadequate statistical techniques according to the distribution of the data), the aim of the present study was to evaluate whether the rally length in high-level volleyball was longer in women than in men. We hypothesised that the mean and median rally duration would be slightly longer in women than in men, and that this difference between genders would be smaller or larger depending on the quantile analysed (with an even smaller difference in short rallies and a larger difference in long rallies). This research attempted to overcome the exposed limitations and provide volleyball coaches and physical trainers with reference values to program the duration of their training series.

## Methods

### Participants

A total of 1,786 rallies were observed: 792 (44.3%) from the Copa de SM La Reina 2020 (the 2^nd^ most important senior women’s volleyball competition in Spain) and 994 (55.7%) from the Copa de SM El Rey 2020 (the 2^nd^ most important senior men’s volleyball competition in Spain). In both knockout tournaments, five matches were played featuring the top six teams from the first round of the regular season of the Spanish Superliga for women and men (Table 1). Teams that finished first and second in the first round of the regular season did not play in the quarter-finals and advanced directly to the semi-finals. Eight rallies (0.4%) were excluded from statistical analysis because a fault was committed before or during the serve execution (i.e., three foot faults of the server, three positional faults of the receiving team, and two misconduct penalties or red cards). In 64 rallies (3.6%), the ball was lost from sight at the end of the rally due to the characteristics of filming. These last rallies were considered *right-censored* or *incomplete observations* and were only analysed using survival analysis statistical techniques (Kaplan and Meier, 1958; Lagakos, 1979).

**Table 1:** Matches and results of the two competitions analysed.

Competition	Match	Teams	Final result	Score of each set
Copa de SM La Reina 2022 (women)	Quarter-final 1	Feel Volley Alcobendas vs. IBSA CV CCO 7 Palmas	3–2	25–21, 23–25, 18–25, 25–19, 15–12
	Quarter-final 2	Sanaya Libby’s La Laguna vs. OSACC Haro Rioja Vóley	2–3	25–22, 21–25, 25–19, 23–25, 13–15
	Semi-final 1	Avarca de Menorca vs. OSACC Haro Rioja Vóley	3–0	25–20, 25–20, 25–20
	Semi-final 2	May Deco Voleibol Logroño vs. Feel Volley Alcobendas	3–0	25–16, 25–16, 25–12
	Final	Avarca de Menorca vs. May Deco Voleibol Logroño	0–3	12–25, 13–25, 17–25
Copa de SM El Rey 2022 (men)	Quarter-final 1	Arenal Emevé vs. Ushuaïa Ibiza Vóley	2–3	23–25, 25–23, 25–22, 15–25, 16–18
	Quarter-final 2	Urbia Vóley Palma vs. UBE L’Illa Grau	2–3	25–21, 24–26, 25–23, 25–27, 12–15
	Semi-final 1	Unicaja Costa de Almería vs. Ushuaïa Ibiza Vóley	3–2	25–22, 22–25, 19–25, 26–24, 17–15
	Semi-final 2	CV Teruel vs. UBE L’Illa Grau	3–0	25–17, 25–18, 25–11
	Final	Unicaja Costa de Almería vs. CV Teruel	2–3	24–26, 25–20, 20–25, 25–20, 13–15
Total	10 matches	12 teams	42 sets	1,786 rallies

### Design and Procedures

A punctual, nomothetic, and multidimensional observational design (Anguera et al., 2011) was used to compare the rally length between women and men. The two competitions analysed were recorded with two digital video cameras fixed on tripods. One camera was placed in the middle of the lateral stand and in line with the net, and the other was placed in the middle of the back stand. All matches were saved in separate video files with a frame rate of 30 FPS, and were played with the sports video analysis software Kinovea v. 0.9.5 (Joan Chartman and contributors, Free Software Foundation, Inc., Boston, MA). This free and open-source software allowed us to open two playback screens, synchronise videos, and insert a stopwatch to measure the rally length in seconds. All rallies were observed from the lateral view or, failing that, from the anteroposterior view. The data obtained were type IV (i.e., concurrent and time-based data) (Anguera et al., 2011). These data were recorded in a spreadsheet with macros created in the Microsoft Excel 2019 program (Microsoft Corp., Redmond, WA). Variables recorded were as follows: (a) *match* (1 = quarter-final 1, 2 = quarter-final 2, 3 = semi-final 1, 4 = semi-final 2, 5 = final); (b) *gender* (0 = men, 1 = women); (c) *rally length* (s = seconds); (d) *pseudo-rally* (0 = no, 1 = yes); and (e) *terminal event* (0 = ball out of sight or right-censored observation, 1 = ball in/out or main event, 2 = fault or competitive event in survival analysis terminology). The rally length was timed from when the server hit the ball until the terminal event occurred (i.e., when the ball touched the floor, when a player touched the net, etc.).

To check the reliability of the data, two observers with a volleyball coaching licence pre-recorded 292 rallies randomly selected from the total sample: 132 from the Copa de SM La Reina 2022 (16.7% of the total women’s sample) and 160 from the Copa de SM El Rey 2022 (16.1% of the total men’s sample). The first observer recorded the 292 rallies twice, two weeks apart. The second observer recorded them only once. Before recording these rallies, both observers received a 2-hour training session to master the Kinovea software, the recording instrument, and the values of the variables studied. Agreement between observations was measured using the Cohen’s *kappa* coefficient (Cohen, 1960) and the Lin’s concordance correlation coefficient (Lin, 1989) for categorical and continuous data, respectively. Intra- and interobserver agreement was greater than 0.98 and 0.93, respectively, for all variables tested. Therefore, all calculated coefficients achieved *excellent* strength of agreement (Byrt, 1996).

### Statistical Analysis

The rally length was represented by a histogram and described by different summary statistics (mean, standard deviation, median, interquartile interval, skewness, and kurtosis).

The assumption of normality of the rally length was checked using P-P plots (standardised normal probability plots). As this assumption was not met, several non-parametric statistical techniques were employed to compare rally length between groups or subsets of data. These techniques included: (a) the Kruskal-Wallis *H* test, followed by pairwise comparisons with Bonferroni correction, to verify if there were differences between matches played within each gender; (b) the Mann-Whitney *U* test to compare the mean rank of both genders; and (c) the bootstrapped quantile regression performed with 100 bootstrap replications (Koenker, 1994) to estimate the effect of gender on rally length from quantile 0.05 to 0.95, including quantile 0.5 or median. The effect size was calculated from the following formulas that included the *z*-value of the Mann-Whitney *U* test (Rosenthal, 1991, 1994): (a) $r=z/\sqrt{N}$; and (b) $d=2\times r/\sqrt{1-r^{2}}$. According to Cohen (1988) and Fritz et al. (2012), effect sizes were interpreted as *small* (*r* = 0.1, *d* = 0.2), *medium* (*r* = 0.3, *d* = 0.5), and *large* (*r* = 0.5, *d* = 0.8).

Moreover, non-parametric survival analysis techniques were applied to evaluate the effect of gender on the *time-to-event* outcome variable (i.e., in our case, the time elapsed until the rally ended by a ball in/out or a fault). The risk or cumulative probability of ending the rally at each time t_j_ was estimated by the multiple decrement model (Gooley et al., 1999) and the Kaplan-Meier method (Kaplan and Meier, 1958). The comparison of the failure curves of both genders was performed using the Pepe and Mori test (Pepe and Mori, 1993) and the log-rank or Mantel-Cox test (Mantel, 1966; Peto and Peto, 1972). The multiple decrement model and the Pepe and Mori test were applied when the terminal event *fault* was considered a competitive event. In contrast, the Kaplan-Meier method and the log-rank test were applied when the terminal event *fault* was not considered a competitive event and was pooled with the main event *ball in/out*.

All statistical analyses were performed with Stata/IC v. 17.0 software (StataCorp, College Station, TX, USA) and repeated for different subsets of data obtained from filtering the original dataset according to the values of the categorical variables of match, gender, pseudo-rally, and terminal event. In these analyses, any difference with a *p*-value less than or equal to 0.05 or with a confidence interval that did not include the value δ = 0 (null hypothesis) was considered statistically significant.

## Results

**Figure 1:**
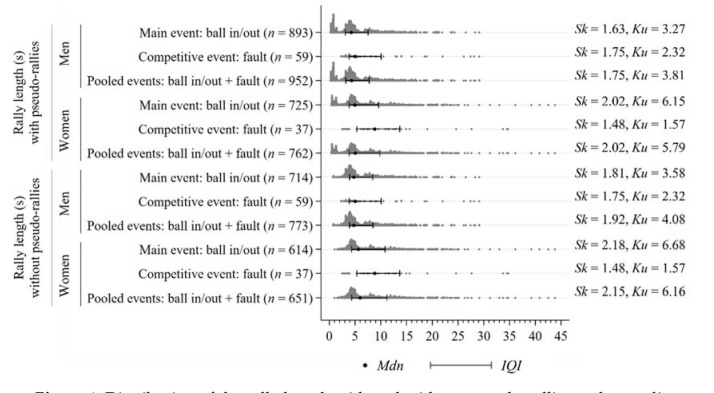
Distribution of the rally length with and without pseudo-rallies and according to gender and terminal events. s = seconds; n = number of rallies; Mdn = median; IQI = interquartile interval; Sk = skewness; Ku = kurtosis

**Figure 2:**
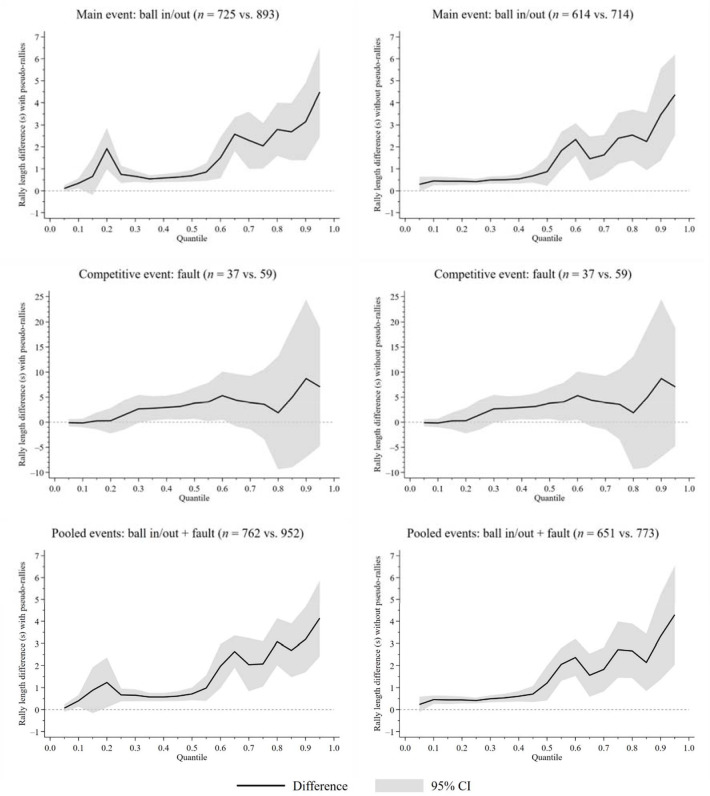
Rally length difference between women and men (reference group) from quantile 0.05 to 0.95. n = number of rallies (women vs. men); s = seconds; CI = confidence interval of the difference between two quantiles obtained from bootstrapped quantile regression

**Figure 3:**
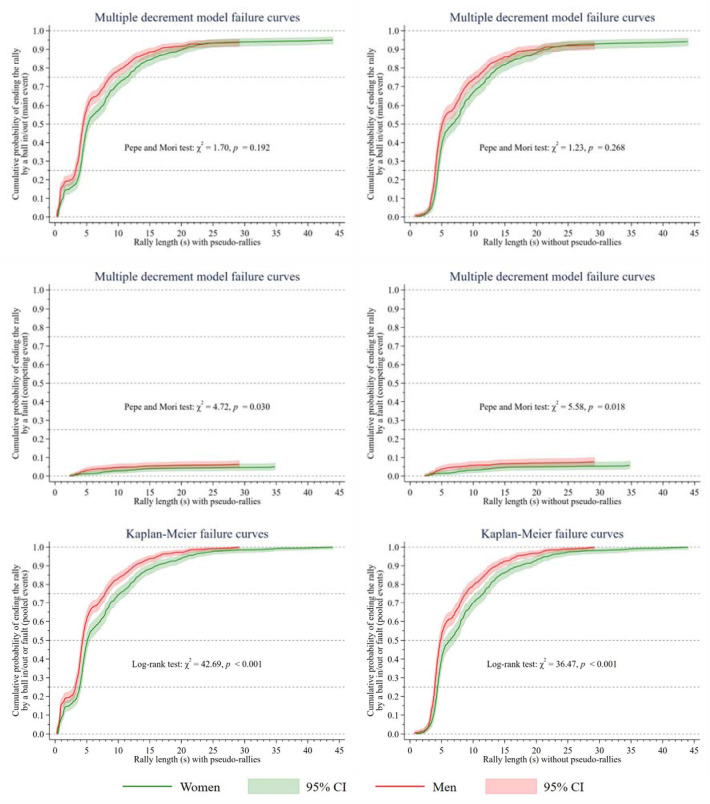
Comparison of the failure curves of women and men. In the left-hand graphs, 791 women’s rallies vs. 987 men’s rallies were analysed. In the right-hand graphs, 680 women’s rallies vs. 808 men’s rallies were analysed. χ^2^ = chi-square statistic; p = p-value of the Pepe and Mori test or the log-rank test; s = seconds; CI = confidence interval of the cumulative probability of occurrence of the event

Analyses of a total of 1,714 rallies (1,424 without pseudo-rallies) are presented in Tables 2 and 3, and Figures 1 and 2. In contrast, analyses of a total of 1,778 rallies (1,488 without pseudo-rallies) are shown in Figure 3. This difference was due to the inclusion or exclusion of 64 right-censored or incomplete observations depending on the type of analysis.

**Table 2:** Mean and median rally length in each match of the two competitions analysed.

	Women			Men		
	*n*	*M* (*SD*)	*Mdn* [*IQI*]	*n*	*M* (*SD*)	*Mdn* [*IQI*]
Rally length (s) with pseudo-rallies						
Quarter-final 1	203	7.03 (6.13)	4.83 [3.70, 9.30]	210	5.99 (5.06)	4.50 [3.40, 8.07]
Quarter-final 2	205	7.06 (6.79)	4.73 [3.57, 8.80]	211	6.32 (5.35)	4.40 [3.36, 8.36]
Semi-final 1	132	7.97 (6.20)	5.32 [4.06, 10.40]	207	5.51 (4.52)	4.28 [3.16, 7.32]
Semi-final 2	111	7.09 (5.62)	4.87 [3.87, 9.37]	117	5.68 (4.99)	4.28 [2.38, 7.70]
Final	111	8.72 (7.12)	7.47 [4.27, 11.90]	207	5.22 (4.42)	4.12 [2.84, 6.80]
						
Rally length (s) without pseudo-rallies						
Quarter-final 1	171	8.16 (6.04)	5.47 [4.33, 10.93]	171	7.17 (4.89)	4.88 [4.00, 8.76]
Quarter-final 2	168	8.41 (6.79)	5.23 [4.30, 10.66]	181	7.25 (5.23)	4.72 [3.88, 9.10]
Semi-final 1	118	8.80 (6.03)	6.17 [4.39, 11.37]	168	6.59 (4.35)	4.70 [3.92, 8.10]
Semi-final 2	97	7.96 (5.47)	5.23 [4.17, 10.28]	92	7.01 (4.84)	5.16 [3.85, 8.59]
Final	97	9.85 (6.92)	8.17 [5.00, 12.48]	161	6.50 (4.22)	4.60 [3.96, 7.86]

s = seconds; n = number of rallies; M = mean; SD = standard deviation; Mdn = median; IQI = interquartile interval

**Table 3:** Comparison of mean and median rally length between women and men (reference group).

	Women		Men		Difference	
	*M* (*SD*)	*Mdn* [*IQI*]	*M* (*SD*)	*Mdn* [*IQI*]	*M* (*p, r*)	*Mdn* [95% CI]
Rally length (s) with pseudo-rallies						
Main event: ball in/out (*n* = 725 vs. 893)	7.25 (6.20)	4.97 [3.87, 9.57]	5.59 (4.67)	4.28 [3.12, 7.52]	1.66 (< 0.001, 0.16)	0.69 [0.42, 0.95]
Competitive event: fault (*n* = 37 vs. 59)	11.50 (9.00)	8.83 [5.37, 13.67]	8.25 (6.95)	5.03 [3.88, 10.08]	3.25 (0.049, 0.20)	3.80 [0.69, 6.91]
Pooled events: ball in/out + fault (*n* = 762 vs. 952)	7.45 (6.42)	5.03 [3.87, 9.83]	5.75 (4.88)	4.32 [3.20, 7.76]	1.70 (< 0.001, 0.16)	0.71 [0.43, 1.00]

Rally length (s) without pseudo-rallies						
Main event: ball in/out (*n* = 614 vs. 714)	8.38 (6.07)	5.63 [4.33, 10.83]	6.79 (4.47)	4.76 [3.92, 8.44]	1.59 (< 0.001, 0.17)	0.87 [0.22, 1.52]
Competitive event: fault (*n* = 37 vs. 59)	11.50 (9.00)	8.83 [5.37, 13.67]	8.25 (6.95)	5.03 [3.88, 10.08]	3.25 (0.049, 0.20)	3.80 [0.69, 6.91]
Pooled events: ball in/out + fault (*n* = 651 vs. 773)	8.56 (6.31)	5.97 [4.33, 11.20]	6.90 (4.72)	4.76 [3.92, 8.48]	1.66 (< 0.001, 0.17)	1.21 [0.42, 2.00]

s = seconds; n = number of rallies (women vs. men); M = mean; SD = standard deviation; Mdn = median; IQI = interquartile interval; p = p-value of the Mann-Whitney U test; r = effect size measure r; CI = confidence interval of the difference between two medians obtained from bootstrapped quantile regression

Table 2 displays the mean and median rally length for each match of the two knockout tournaments analysed. The results are presented both with and without pseudo-rallies. Based on the Kruskal-Wallis *H* test, no significant differences were found between the men’s matches (*p* = 0.336 with pseudo-rallies, *p* = 0.753 without pseudo-rallies). However, significant differences were found among the women’s matches (*p* = 0.024 with pseudo-rallies, *p* = 0.040 without pseudo-rallies). Specifically, significant differences were found in three pairwise comparisons out of a total of 10 (quarter-final 1 vs. final, quarter-final 2 vs. final, and semi-final 2 vs. final).

Figure 1 shows the distribution of the continuous variable rally length according to the values of the categorical variables of gender, pseudo-rally, and terminal event. The rally length presented a positive skewness (right-skewed distribution) and a positive kurtosis (leptokurtic distribution). This positive skewness and kurtosis were greater in women when the rally ended with a ball in/out, while in men, these values were greater when the rally ended with a fault. Furthermore, the rally length presented a higher range, interquartile range, and maximum value in women. Thus, these descriptive measures indicated a greater length and dispersion in women’s rallies.

Table 3 shows that the mean and median rally length was longer in women than in men. The Mann-Whitney *U* test and the effect size measures revealed significant and small differences in rally length between the two genders (*p* < 0.05, *r* = 0.16‒0.20, *d* = 0.32‒0.41). These differences were slightly larger when pseudo-rallies were excluded from the analysis (*r* = 0.16, *d* = 0.32 with pseudo-rallies vs. *r* = 0.17, *d* = 0.34 without pseudo-rallies); and they were greater when the rally ended with a fault (*r* = 0.16‒0.17, *d* = 0.32‒0.34 when it ended with a ball in/out vs. *r* = 0.20, *d* = 0.41 when it ended with a fault). The differences between the medians—which were statistically significant because their confidence intervals did not include the value δ = 0—confirmed these results. In specific relation to pseudo-rallies, fewer serve errors and aces were observed in women than in men, both in absolute (111 vs. 179) and relative terms (14.0% vs. 18.1%, percentage difference = −4.1%, 95% CI [−7.5%, −0.7%], *p* = 0.020 in the Wald test). The mean duration of pseudo-rallies was 0.95 s (*SD* = 0.36) and 0.79 s (*SD* = 0.33) in women and men, respectively (mean difference = 0.16 s, *p* = 0.002 in the Mann-Whitney *U* test, *r* = 0.18, *d* = 0.37); and the median duration of pseudo-rallies was 0.90 s (*IQI* [0.60, 1.33]) and 0.76 s (*IQI* [0.56, 0.90]) in women and men, respectively (median difference = 0.14 s, 95% CI [0.01, 0.27] in the bootstrapped quantile regression).

Figure 2 shows the rally length difference between genders in different quantiles. The results in quantile 0.5 of this figure coincide exactly with the results of the last column of Table 3. On the other hand, results in the rest of the quantiles provide new information. Interestingly, when the rally ended with a fault, significant differences between genders were only observed from quantile 0.35 to 0.6. In contrast, when the rally ended with a ball in/out or when the terminal events ball in/out and fault were pooled, significant differences were observed in virtually all quantiles, with the exception of quantiles 0.05 and 0.15—the latter, only in the analysis with pseudo-rallies. Specifically, with the terminal events pooled and at the descriptive level (see the black solid lines in Figure 2), the rally length difference between genders was barely 1 s in quantile 0.5 or median, while in quantile 0.95, it was just over 4 s—always in favour of women. Furthermore, at the inferential level (see grey bands in Figure 2), it was found that between quantiles 0.5 and 0.95, this difference increased significantly, as the limits of their confidence intervals did not overlap. The results tables that were used to construct the graphs in Figure 2 are presented in detail in Appendix A.

Finally, Figure 3 shows that the men’s failure curve (red) grew faster than the women’s (green). This indicated a worse survival in game continuity in men. Regardless of the inclusion or exclusion of pseudo-rallies, no significant differences (*p* > 0.05) were found between genders when the rally ended with a ball in/out. In contrast, significant differences (*p* ≤ 0.05) were found when the rally ended with a fault or when the terminal events ball in/out and fault were pooled. In the latter case, the confidence intervals of the curves did not overlap from second 0 to 1 (with pseudo-rallies) and from second 4 to 19 (with and without pseudo-rallies). Therefore, statistically significant differences were found between women and men in these time intervals. In women, the probability of ending the rally at 3.9, 5.1, 10.2, and 43.9 s (at 4.4, 6.3, 11.6, and 43.9 s without pseudo-rallies) was 25%, 50%, 75%, and 100%, respectively. In men, the probability of ending the rally at 3.2, 4.3, 7.9, and 29.1 s (at 3.9, 4.8, 8.8, and 29.1 s without pseudo-rallies) was 25%, 50%, 75%, and 100%, respectively. The results tables that were used to construct the graphs in Figure 3 are presented in detail in Appendix B.

## Discussion

The aim of the present study was to evaluate whether the rally length in high-level volleyball was longer in women than in men. To achieve this objective and confirm the hypotheses of the study, two volleyball competitions were analysed: (a) the Copa de SM La Reina 2020 and (b) the Copa de SM El Rey 2020. Both competitions were comparable because, with the exception of gender, they had the same characteristics (i.e., same country, season, age category, level of play, type of tournament, number of participating teams, and number of matches played). As shown in Tables 1 and 2, there was greater equality in the men’s tournament compared to the women’s tournament. Four tiebreaks were played in the men’s tournament and no significant differences were found when comparing the central tendency of rally length across the five matches played. In contrast, in the women’s tournament, only two tiebreaks were played, and significant differences were found in the central tendency of rally length between three pairwise comparisons (quarter-final 1 vs. final, quarter-final 2 vs. final, and semi-final 2 vs. final). The reason for this fact is unknown. However, it highlights the need to consider the competitive context in any study of volleyball that analyses the technical-tactical behaviour and performance of teams and players. In this regard, Lago-Peñas (2012) suggests that contextual or situational variables, such as the quality of the opposition, should be thoroughly investigated to understand their influence in team sports.

To our knowledge, the vast majority of previous studies on rally length in volleyball have analysed women and men of the highest national or international level separately (Aytar et al., 2019; García-de-Alcaraz et al., 2017; Sánchez-Moreno et al., 2015, 2016), and only few studies have analysed both genders together, but in sitting volleyball (Tsakiri et al., 2021) or without specifying the methodology applied to record and analyse the data (FIVB, 2019). This common problem of analysing both genders separately in standing volleyball, together with other methodological problems that will be discussed below, seriously hindered the contrasting of our results with other similar studies. In this sense, one of the most serious problems found in all the studies consulted was that the rally length was described from the mean and the standard deviation, despite the fact that this continuous variable did not follow a normal distribution and that it presented a marked positive skewness. In our case, precisely to be able to compare results, we were forced to also calculate the mean and standard deviation, although we believe that, based on the distribution of the data, it is more appropriate to calculate other more robust measures of central tendency and dispersion, such as the median and interquartile interval (Streiner, 2000).

As a key point, in the present study the important decision was also made to measure the rally length in seconds from the exact moment the server hit the ball to the exact moment the event that ended the rally occurred (i.e., when the ball touched the floor, when a player touched the net, etc.). The reason for this decision is that this way of measuring the rally length was considered more precise and accurate than that used in other previous studies. On the one hand, Aytar et al. (2019) and Sánchez-Moreno et al. (2015, 2016) timed the rally from the hit of the server to the last hit before the end of the rally (e.g., the last spike), which shortened the rally length. Possibly for this reason, the mean rally length was shorter in these studies (6.88 s for Turkish high-level women and 4.99 s for international high-level men, without specifying the inclusion or exclusion of pseudo-rallies) than in ours (7.45 s for Spanish high-level women and 5.75 s for Spanish high-level men, including pseudo-rallies). On the other hand, García-de-Alcaraz et al. (2017) timed the rally—as indicated in the official volleyball rules (FIVB, 2021)—from the hit of the server until the referee manually signalled that the ball was out of play, which increased the rally length. Probably for this reason, the mean rally length was longer in that study (5.84 s for Spanish high-level men and 6.79 s for international high-level men, without specifying the inclusion or exclusion of pseudo-rallies) than in ours (5.75 s for Spanish high-level men, including pseudo-rallies).

In addition to the way the rally length is measured, other methodological issues that made it difficult to contrast results were that not all previous studies analysed the same level of play or the same country, and that no study—with the exception of the FIVB *Picture of the Game* annual report (2019)—specified whether or not they included pseudo-rallies in their analysis. Interestingly, our results (mean rally length with pseudo-rallies = 7.45 s in women and 5.75 s in men; mean rally length without pseudo-rallies = 8.56 s in women and 6.90 s in men) were very similar to those published in the FIVB report (mean rally length with pseudo-rallies = 7.13 s in women and 5.65 s in men; mean rally length without pseudo-rallies = 8.31 s in women and 7.10 s in men), with a difference of 1 to 3 tenths. However, those results are not considered comparable because an international competition was analysed in the FIVB report (2019 FIVB Volleyball Nations League final round) and because the methodology used to time the rallies was not specified in this report.

In relation to the study’s hypothesis, it was found that the mean and median rally length was significantly and slightly longer in women than in men, regardless of how the rally ended (ball in/out or fault) or the inclusion/exclusion of pseudo-rallies. Consequently, these findings confirm our initial hypothesis that women’s rallies last slightly longer in central values than men’s. However, if we only compare the mean and median of both genders, these findings are very limited, as they ignore whether this *small* difference is larger or smaller (or even non-significant) in other quantiles lower or higher than quantile 0.5 or median. And so it was in our case, since, for example, when comparing quantile 0.95 (i.e., when comparing long rallies), a non-significant difference was found between women and men when the rallies ended with a fault; in contrast, when the rallies ended with a ball in/out or when the terminal events ball in/out and fault were pooled, a significant and larger difference was observed than that found in quantile 0.5, since the difference increased from 1 to 4 s approximately. The latter confirms our hypothesis that the difference between genders may vary depending on the quantile analysed.

According to João et al. (2010), the fact that the rally length is longer in women than in men may be due to other differences between them, such as their anthropometric and physiological differences (i.e., men are taller, jump higher, have a higher centre of gravity, etc.). The fact is that, despite the height of the net being higher in men (2.43 m vs. 2.24 m), several authors such as Kountouris et al. (2015) and Lima et al. (2019) argue that men’s volleyball is more associated with terminal actions (serve errors and aces, power jump serves, hard-driven spikes, and kill blocks), while women’s volleyball is more associated with continuity actions (off-speed spikes and effective defences). Proof of this is that, in the present study, more serve errors and aces were observed in men than women (18.1% vs. 14.0%), which indicates that men assume higher risk when serving (Lima et al., 2019). And if men risk more at the beginning of each rally with the serve, committing more errors and making more aces, it is logical to think that they also risk more at any other moment of the game with other terminal actions such as the attack/counterattack or the block, committing more unforced errors or faults that cause rallies to end prematurely. In this respect, in our study, it was also observed that men committed more faults than women (6.2% vs. 4.9% considering pseudo-rallies).

Regarding the results obtained in the survival analysis, these also confirmed our hypothesis (i.e., men had a worse survival in game continuity than women) and provided complementary information of practical use for volleyball coaches and physical trainers. Specifically, the cumulative probability of ending the rally at each time t_j_ plotted on the failure curves in Figure 3 is considered useful for programming volleyball-specific resistance training, as long as the coach or physical trainer wants to reproduce in training the physical demands of competition. For example, in men, without pseudo-rallies, and with the terminal events pooled, if a physical trainer of a high-level Spanish team prepares a 12-station circuit on an 18 × 9 m volleyball playing court (six stations in each 9 × 9 m half-court), in which specific volleyball movements without a ball are performed at each station (e.g., transitions from blocking to spiking in front zones, and defensive movements and emergency digs in back zones), then players should randomly and explosively complete three stations (25% of the total) between 0.8 and 3.9 s of work, three stations between 4.0 and 4.8 s, three stations between 4.9 and 8.8 s, and three stations between 8.9 and 29.1 s—with a mean rest time between stations of approximately 27.4 s, obtained from the results of García-de-Alcaraz et al. (2017) in the first Spanish senior division.

Despite the strengths of the present study, related to considering pseudo-rallies and the event that ends the rally, proposing a specific method to measure rally length applied to both women and men, and using advanced non-parametric statistical techniques to analyse the data, there were also a number of limitations that are presented below.

First, it is important to note that, having analysed only two Spanish competitions, the results obtained in this study cannot initially be extrapolated to other lower-level Spanish competitions or to other competitions of the same level held in other countries. Therefore, in future studies, it is suggested to analyse other countries, age categories, levels of play, types of tournaments, and even other sports disciplines recognised by the FIVB, such as 2 × 2 beach volleyball (Olympic sport since 1996) and 3 × 3 snow volleyball (demonstration sport at the 2018 Winter Olympic Games). Considering beach volleyball, there are already studies that have analysed the mean rally length (FIVB, 2015; Medeiros et al., 2014; Palao et al., 2014, 2015).

Second, in the design and planning phase of this study, the minimum number of rallies per gender needed to compare two means was not calculated, because no previous scientific studies were found that jointly analysed both genders in a similar competition. However, based on, for example, the results presented in the third row of Table 3 (i.e., rally length with pseudo-rallies and with the terminal events pooled), it is possible to estimate the sample size needed for future studies. Indeed, in a one-sided test, accepting an alpha risk of 5% and a beta risk of 10%, assuming a *n*_0_/*n*_1_ ratio of 1.25 and a common standard deviation of 5.62, and anticipating a dropout rate of 4%, 176 rallies would be necessary in the women’s group and 220 in the men’s group to recognize as statistically significant a rally length mean difference greater than or equal to 1.70 s.

Third, despite having filmed the matches from two views (lateral and anteroposterior), 64 rallies could not be fully timed, thus being considered incomplete or right-censored observations. These rallies could only be analysed using statistical survival analysis techniques and not using more common non-parametric techniques, such as the Mann-Whitney *U* test.

Fourth, the videos analysed had a frame rate of only 30 FPS, which meant a time of 0.03 s between frames. Therefore, in future studies, it is recommended to analyse videos recorded at 60 FPS or more, in order to increase the accuracy of the Kinovea software stopwatch.

Fifth, timing each rally with the Kinovea software stopwatch—a stopwatch previously validated in biomechanical and kinematic studies (Balsalobre-Fernández et al., 2014; Fernández-González et al., 2022)—required a lot of time and dedication, as each rally was played in slow motion or frame-by-frame as needed. For this reason, in future studies, it is recommended to use another sports video analysis software that is more agile than Kinovea, which allows timing rallies in real time and recording more data in less time. In this regard, in professional volleyball, there is scouting software used by the best teams in the world, called Data Volley (Data Project - Genius Sports Media, Los Angeles, CA), which makes it possible to time rallies in real time. However, before using the stopwatch of this software, it would be recommended to validate it.

Sixth, the rally length was analysed without considering the possible effect of contextual variables such as the quality of opposition (low, intermediate, high), the match location (home, away, neutral), the type of the set (initial, final), the period within the set (beginning, end), the match status (high disadvantage, moderate disadvantage, balanced, moderate advantage, high advantage), or match type (balanced, unbalanced). Some of these situational variables have been previously analysed in volleyball studies, which investigated their influence on the technical-tactical performance of certain actions (Marcelino et al., 2011, 2012) or game roles (García-de-Alcaraz and Usero, 2019). However, these types of variables have commonly been studied more extensively in other team sports such as soccer (Fernandes et al., 2020; Lago-Peñas, 2012). In our study, due to the characteristics of the competitions analysed (knockout tournaments played in a neutral location and with the six best ranked teams in the first round of the women’s and men’s Spanish Superliga), the effect of some of these contextual variables was neutralized to some extent. However, in future studies of rally length, it is recommended to incorporate these variables with a larger sample of matches and teams. In this way, it will be possible to verify new and interesting hypotheses, such as, for example, that the rally length is greater when the level of teams is equal (low vs. low, intermediate vs. intermediate, or high vs. high) than when it is unequal (low vs. intermediate, low vs. high, intermediate vs. high).

## Conclusions

This study highlights that in high-level Spanish volleyball, the mean and median rally length was significantly and slightly longer in women than in men, regardless of how the rally ended or the inclusion/exclusion of pseudo-rallies. At other quantiles below or above quantile 0.5 or median, this gender difference varied and was smaller or even larger. Survival analysis confirmed these results and also provided a series of time thresholds that volleyball coaches and physical trainers can use in training to prepare players coherently, without neglecting the physical demands of competition. Due to the fact that the continuous variable rally length presented a marked positive skewness and did not meet the assumption of normality, in future studies it is recommended to describe it using the median and the interquartile interval, and analyse it using advanced non-parametric statistical techniques, such as quantile regression.

## Appendices

Graphs in Figures 2 and 3 were created from very extensive results tables that can be found in Appendix A (https://rhileno.shinyapps.io/rally_length_volleyball_appendix_A/) and Appendix B (https://rhileno.shinyapps.io/rally_length_volleyball_appendix_B/), respectively.

For space and practical reasons, these two appendices are built from Shiny version 1.7.4 (R package for building interactive web applications) and R Studio integrated development environment version 2021.09.0 (RStudio, PBC, Boston, MA).
